# The Microbiota in Cancer: A Secondary Player or a Protagonist?

**DOI:** 10.3390/cimb46080463

**Published:** 2024-07-23

**Authors:** Ana María Gómez García, Francisco López Muñoz, Eduardo García-Rico

**Affiliations:** 1 Internal Medicine Unit, Hospital Universitario HM Madrid, 28015 Madrid, Spain; anagomezgarcia@empleado.hmhospitales.com; 2 Facultad HM de Ciencias de la Salud de la Universidad Camilo José Cela, 28692 Madrid, Spain; flopez@ucjc.edu; 3 Instituto de Investigación Sanitaria HM Hospitales, 28015 Madrid, Spain; 4 Medical Oncology Unit, Hospital Universitario HM Torrelodones, 28250 Torrelodones, Spain

**Keywords:** microbiota, cancer, dysbiosis, cancer microbiota, PD-1, PDL-1, immunotherapy, allostatic load

## Abstract

The intestinal microbiota and the human body are in a permanent interaction. There is a symbiotic relationship in which the microbiota plays a vitally important role in the performance of numerous functions, including digestion, metabolism, the development of lymphoid tissue, defensive functions, and other processes. It is a true metabolic organ essential for life and has potential involvement in various pathological states, including cancer and pathologies other than those of a digestive nature. A growing topic of great interest for its implications is the relationship between the microbiota and cancer. Dysbiosis plays a role in oncogenesis, tumor progression, and even the response to cancer treatment. The effect of the microbiota on tumor development goes beyond a local effect having a systemic effect. Another aspect of great interest regarding the intestinal microbiota is its relationship with drugs, modifying their activity. There is increasing evidence that the microbiota influences the therapeutic activity and side effects of antineoplastic drugs and also modulates the response of several tumors to antineoplastic therapy through immunological circuits. These data suggest the manipulation of the microbiota as a possible adjuvant to improve oncological treatment. Is it possible to manipulate the microbiota for therapeutic purposes?

## 1. Introduction

We call the microbiota the set of species, whether bacterial, archaeal, fungal, or viral, that coexist in a non-pathological way in an organism. We use the term microbiome in reference to the set of genes of these species.

The complex interactions between these microbial communities and the host immune responses are fundamental to maintaining homeostasis and overall health. The human body and the intestinal microbiota engage in a continual, millennia-old interaction crucial for human health [1,2,3]. Formerly perceived as a symbiotic relationship, recent insights have unveiled its complexity, highlighting the microbiota’s pivotal role in various bodily functions [4]. Indeed, it is now recognized as a distinct organ, wielding specific functions vital for health maintenance: a microcosm within us, significantly influencing our physiology and pathophysiology [5,6,7,8]. The complex interactions between these microbial communities and the host immune responses are fundamental to maintaining homeostasis and overall health. The microbiota plays a crucial role in digestion, essential for proper nutrition and bodily growth. For instance, studies on germ-free mice, i.e., in the absence of intestinal microbiota, reveal lower body weight, reduced vital organ size (anomalous and deficient development of the heart, liver, and lungs), decreased adiposity, and an underdeveloped immune system with decreased Ig (immunoglobulin) levels [9,10]. Moreover, it contributes to metabolism by fermenting indigestible dietary waste and endogenous mucus, boasting 80 families of glucose-hydrolases capable of degrading non-digestible fiber, a process not achievable by humans alone.

It aids in the development of lymphoid tissue associated with the intestine, thereby supporting immune system integrity. Additionally, it metabolizes bile acids and xenobiotics and synthesizes vitamins B and K, and its antigens and metabolic byproducts stimulate cytokine production against potential pathogens [11,12].

It serves a dual purpose: defensively, it acts as a barrier against pathogens, while trophically, it regulates the proliferation and differentiation of epithelial cells. Furthermore, numerous molecules produced by the intestinal microbiota exhibit immunomodulatory activity, including oligonucleotides, peptides, proteins, short-chain fatty acids, lipopolysaccharides, peptidoglycans, choline degradation products, and endocannabinoids [13,14,15].

In recent years, increasing scientific evidence has revealed its possible involvement in various pathologies beyond digestive disorders, including cancer [16,17,18]. In addition, the microbiota, due to its complexity and functional interconnections, is beginning to be considered to constitute a brain–gut axis mediated by the vagus nerve (parasympathetic system) and sympathetic and neuroendocrine systems associated with the gastrointestinal tract [19,20]. Today, we know that bacterial intestinal colonization is essential for the proper maturation of this hypothalamic–pituitary–adrenal (neuroendocrine) axis (HPA) [21].

Numerous diseases have been associated with alterations in the gut microbiota, such as obesity, diabetes, inflammatory bowel disease, and even central nervous system disorders such as autism, anxiety, depression, and alcohol dependence [22,23,24,25,26,27,28,29,30,31]. A seminal study by Strachan in 1989 highlighted the correlation between decreased microbial exposure due to increased hygiene in developed countries and the increased prevalence of autoimmune diseases, probably attributable to the modulatory or regulatory effects of the microbiota on the immune response [32,33,34].

An imbalance in the microbial communities residing in the human body, especially in the gut, is referred to as dysbiosis. Under physiological conditions, a healthy microbiome, known as eubiosis, plays a crucial role in digestion, immune function, and the synthesis of vital nutrients. The balance of these microbial populations is essential for maintaining homeostasis and overall health. When this balance is disturbed, dysbiosis occurs, which may be due to factors such as inadequate diet, antibiotic use, and stress.

In pathological conditions, dysbiosis is associated with various diseases. It has been associated with gastrointestinal disorders, such as inflammatory bowel disease (IBD) and irritable bowel syndrome (IBS), as well as systemic conditions, such as obesity, type 1 and 2 diabetes, and even neurological disorders, such as autism and depression. Alterations in the gut microbiota can alter immune responses and metabolic pathways, exacerbating the symptoms and progression of these diseases.

The concept of “eubiosis” can best be understood as a form of allostasis, i.e., the process by which the organism maintains stability through change, especially in response to stress. Allostatic load represents the cumulative burden of chronic stress and life events on the body’s physiological systems, resulting in wear and tear and increased susceptibility to disease. The gut microbiota plays a crucial role in this context, as it interacts with the endocrine, immune, and nervous systems of the host, as previously mentioned. These interactions influence the body’s responses to stress and overall homeostasis. The aforementioned gut–brain axis facilitates this interaction. Microbial metabolites can modulate the hypothalamic–pituitary axis [35].

Recent research highlights that dysbiosis, or gut microbiota imbalance, is associated with increased allostatic load. This condition is characterized by hypercortisolemia, chronic inflammation, and impaired regulation of the HPA axis. In contrast, a balanced microbiome can improve stress resistance by promoting anti-inflammatory pathways and maintaining the integrity of the intestinal barrier. Interventions such as dietary modification, probiotics, and physical activity can positively influence the gut microbiome, thereby reducing allostatic load and improving stress resistance. For example, physical activity has been shown to increase microbial diversity and the presence of beneficial bacteria, which contribute to improved physiological responses to stress [36,37].

The aim of this review is to provide an overview of the microbiota, especially focusing on the positive and negative interactions between the microbiome and the host organism in relation to cancer. To this end, we have not only reviewed those situations in which the microbiota is directly responsible for the disease but also especially highlighted those situations in which the genesis of cancer is mediated by the immune system. In the latter case, we also reviewed the role of the microbiota in modulating the action of new onco-immunological therapies, such as checkpoint inhibitors, used in lung cancer, colon cancer, melanoma, and other tumors.

## 2. Composition

The human body hosts several billion microbes, encompassing approximately 500 to 1000 different species, with the majority residing in the digestive tract. Their abundance far surpasses that of human cells, with microbial genes numbering between 5 and 8 million compared to approximately 20,000 human genes [38]. Remarkably, they contribute up to 1–2 kg of body weight. Such statistics underscore that their significance, importance, and health implications extend beyond mere symbiosis.

To deepen our understanding, the National Institutes of Health launched the Human Microbiome Project in 2007, aiming to explore potential correlations between microbiome changes and human health outcomes [38,39]. Advancements in ecosystem knowledge have been propelled by the introduction of mass sequencing techniques targeting the 16S rRNA gene (16S rDNA gene). Previously, microbial flora investigations relied solely on cultivation methods, which were limited as many microbes are non-cultivable [40,41].

The predominant phyla in the human intestinal microbiota include Firmicutes, Bacteroidetes, Actinobacteria, Proteobacteria, Fusobacteria, Verrucomicrobia, Tenericutes, and Lentisphaerae. Additionally, key genera within this ecosystem comprise *Bacteroides*, *Clostridium*, *Faecalibacterium*, *Eubacterium*, *Ruminococcus*, *Peptococcus*, *Peptostreptococcus*, *Lactobacillus*, *Streptococcus*, *Streptomyces*, and *Bifidobacterium* [11,42]. Among adults, Firmicutes (approximately 60%) and Bacteroidetes (approximately 25%) represent the majority of bacterial populations (Table 1).

The composition and diversity of the microbiota are equally important to their abundance. Maintaining balanced proportions is crucial, with the Firmicutes/Bacteroidetes ratio serving as a parameter to evaluate intestinal microbiota balance and functionality. For instance, in individuals with obesity, this ratio is significantly altered due to an increase in Firmicutes [43]. Moreover, a physiological increase in Firmicutes has been observed in the elderly, attributed to aging processes [19,44].

In addition to the balance between Firmicutes and Bacteroidetes, there are other important balances in the intestinal microbiota that, when disturbed, can have significant health consequences. Some of the most relevant imbalances are described here:

Actinobacteria:

Imbalance: An increase or decrease in the proportion of Actinobacteria, particularly the genus *Bifidobacterium*.

Consequences: It may lead to decreased carbohydrate fermentation and short-chain fatty acid (SCFA) production, affecting intestinal health and the intestinal barrier. *Bifidobacterium* depletion has been associated with inflammatory bowel diseases and obesity.

Proteobacteria:

Imbalance: An increase in the proportion of Proteobacteria, which includes genera such as *Escherichia*, *Salmonella*, and *Helicobacter*.

Consequences: An increase in Proteobacteria is often an indicator of dysbiosis and is associated with inflammatory bowel diseases, gastrointestinal infections, and metabolic diseases. These bacteria can cause inflammation and damage to the intestinal mucosa.

Verrucomicrobia:

Imbalance: Changes in the abundance of *Akkermansia muciniphila*, the main representative of this phylum in the gut.

Consequences: A reduction in *Akkermansia* has been linked to metabolic diseases such as obesity and type 2 diabetes. This bacterium plays an important role in mucin degradation and in maintaining the integrity of the intestinal barrier.

Fusobacteria:

Imbalance: An increase in the proportion of *Fusobacterium*.

Consequences: These bacteria are associated with periodontal diseases and certain types of cancer, such as colorectal cancer. *Fusobacterium* can promote biofilm formation and have pathogenic properties.

Cyanobacteria:

Imbalance: Although less common, a change in the abundance of Cyanobacteria in the gut.

Consequences: These organisms may influence photosynthesis and nitrogen fixation, and although their role in the human gut is not completely clear, it is suggested that they may interact with other gut bacteria and affect the microbiota in general.

### Microbiota Acquisition

The acquisition of microbiota commences intrauterinely, contrary to previous beliefs regarding the sterility of this environment. Its origins trace back to the placenta, amniotic fluid, umbilical cord blood, and meconium. During birth, there occurs vertical maternofetal transmission, with the mode of delivery influencing microbiota composition [45,46]. Vaginal delivery exposes the fetus to microorganisms from the birth canal, primarily *Lactobacillus* and *Prevotella*. In contrast, cesarean section delivery exposes the fetus to skin microorganisms, predominantly *Staphylococcus*, *Corynebacterium*, and *Propionibacterium*, resulting in lower *Bifidobacterium* isolation rates and a reduced prevalence of *Bacteroides* [47].

Moreover, the type of lactation impacts newborn colonization, with breastfed infants exhibiting a healthier microbiota compared to their formula-fed counterparts. This is attributed to the presence of lactic acid bacteria and bifidogenic factors in breast milk, promoting increased growth of *Lactobacillus* and *Bifidobacterium* [48,49].

During the early years of life, the introduction of solid foods enriches the microbiome, enhancing its diversity. Consequently, this period plays a pivotal role in shaping an individual’s microbiota, which continues to evolve through interactions between the host and the environment, including factors such as diet, lifestyle, diseases, and antibiotic usage [38]. Once established, the microbiota tends to remain stable over time.

**Figure 1:**
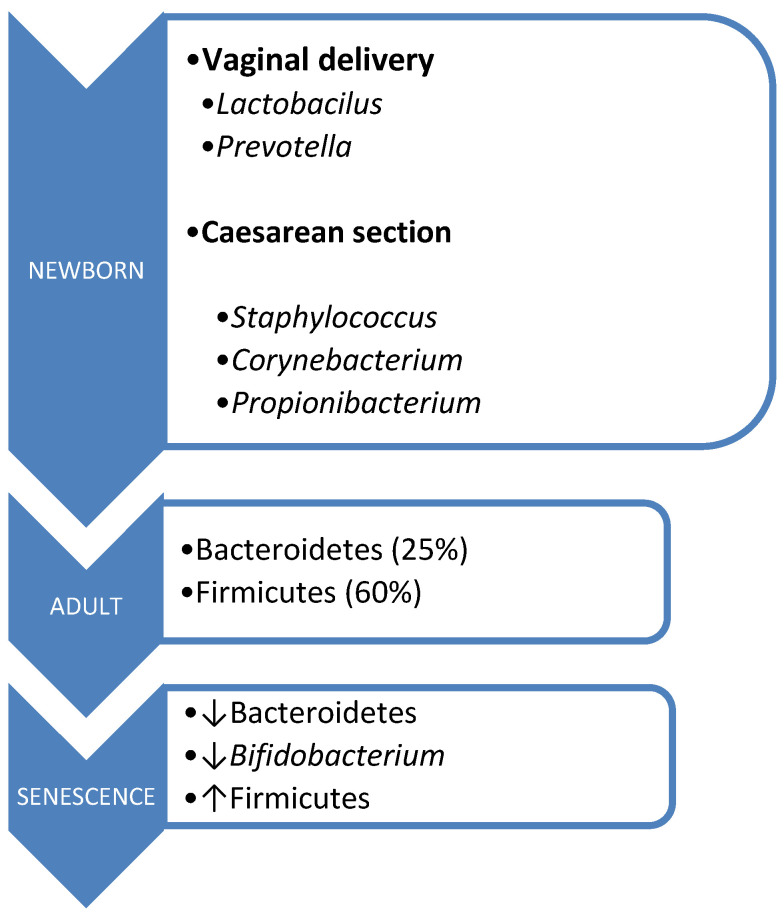
Microbiota composition according to age.

However, aging brings about changes in the composition of the intestinal microbiota, influenced by factors such as immunosenescence, aging of the intestinal mucosa, and alterations in dietary patterns. This is characterized by a decline in Bacteroidetes and *Bifidobacterium* populations, alongside an increase in Firmicutes [38,50] (Figure 1).

## 3. Microbiota and Cancer

### 3.1. Non-Immunologically Mediated Relationship

An increasingly compelling area of research with significant implications is the relationship between the microbiota and cancer [51,52]. Alterations in the interplay among the intestinal flora, the intestinal epithelium, and the immune system are linked to numerous diseases, including cancer [53,54]. Dysbiosis, characterized by an imbalance in the host–intestinal microbiota relationship, contributes to oncogenesis and tumor progression and even impacts the response to cancer treatment [55].

Dysbiosis has been implicated in the etiology of several types of cancer. This imbalance can lead to altered production of microbial metabolites, changes in local and systemic inflammation, and modifications in the immune response. For example, it has been observed that an increase in pro-inflammatory species such as *Escherichia coli* and a decrease in anti-inflammatory bacteria such as *Faecalibacterium prausnitzii* can promote a chronic inflammatory environment in the gut, which is a known risk factor for the development of colorectal cancer [56].

In addition, dysbiosis may influence the efficacy of immunotherapy and chemotherapy in cancer treatment. It has been shown that certain intestinal bacteria can metabolize chemotherapeutic agents, affecting their bioavailability and efficacy. On the other hand, the microbiota can modulate the host immune response, affecting the body’s ability to attack tumor cells. A study by Routy et al. [57] revealed that the presence of *Akkermansia muciniphila* in the gut is associated with a better response to immunotherapy in cancer patients, highlighting the importance of the microbiome in modulating the response to treatment. The gut microbiome influences the efficacy of PD-1-based immunotherapy against epithelial tumors.

Numerous mechanisms contribute to this phenomenon. For instance, hydrogen sulfide, a byproduct of the intestinal flora, and *Bacteroides fragilis* toxin target intestinal epithelial cells [58]. Conversely, short-chain fatty acids, primarily acetic, propionic, and butyric acids—products of complex dietary polysaccharide digestion by microbial enzymes—exhibit anticarcinogenic properties. Butyric acid, in particular, acts as an energy source for colonocytes, thus exerting a protective effect against carcinogenesis [59]. But, the effect of the microbiota on tumor development goes beyond a local effect. It has been shown that the experimental alteration of the intestinal microbiota also influences the incidence and progression of extraintestinal tumors, such as breast and hepatocarcinoma [51], therefore having a systemic effect [56,60,61,62,63].

Established risk factors for cancer, including obesity, tobacco use, and stress, are also acknowledged inducers of dysbiosis, suggesting a potential link between the two [64,65]. Notable instances of microorganisms associated with cancer development include *Helicobacter pylori* in gastric cancer [66,67], *Human papillomavirus* in cervical cancer [68], and *Hepatitis C virus* in liver cancer [69,70] (Table 2).

Recent advancements in ribosomal RNA sequencing of stool samples from patients with colon cancer and healthy individuals have revealed the involvement of bacteria such as *Fusobacterium* in cancer development [71,72]. Dysbiosis induced by these bacteria fosters inflammation and cancer cell proliferation. Conversely, bacteria like *Bifidobacterium* stimulate immunological activity (macrophages, T cells) and confer protection against cancer development, with evidence suggesting their potential to attenuate tumor growth [38,71].

A study published in the Journal of the National Cancer Institute in 2013 [73] compared the microbiota of colon cancer patients to that of healthy individuals, revealing a tendency for cases to exhibit an enrichment of the Bacteroidetes phylum and a depletion of Firmicutes. Within Firmicutes, a notable relative loss was observed, particularly within the *Clostridium* class, including *Coprococcus*. *Clostridium*, particularly *Coprococcus*, efficiently ferment dietary fiber and complex carbohydrates to produce butyrate, a colonic metabolite known to inhibit colonic inflammation and carcinogenesis. It has demonstrated antitumorigenic properties such as inhibiting tumor cell proliferation, inducing tumor cell apoptosis, and modulating the homeostasis of regulatory T cells. The depletion of these bacterial populations, coupled with an enrichment of pathogenic populations, likely synergistically contributes to tumorigenesis [73].

Moreover, the genus *Fusobacterium* was significantly elevated in cases compared to controls and was associated with an increased risk of colon cancer [74,75,76]. Gram-negative anaerobes like *Fusobacterium* contribute to colitis and periodontal disease, which in itself could be related to colorectal cancer [77].

Other studies have reported similar findings, noting an increase in *Fusobacterium* levels on the surface of tumors compared to adjacent healthy tissue, suggesting its potential as a marker for tumor presence. Additionally, enterotoxigenic *Bacteroides fragilis*, a pathogenic variant of a commensal bacterium, has been demonstrated to influence the development of colorectal cancer in murine models through the production of a metalloprotease toxin [78]. Furthermore, the loss of potentially protective bacterial populations also contributes to tumorigenesis. The simultaneous depletion of these protective bacteria, coupled with an enrichment of pathogenic populations, likely synergistically enhances the development of tumors.

To date, colon cancer screening primarily relies on methods such as fecal occult blood testing, which has its limitations, and colonoscopy, which is invasive, carries risks, and entails substantial costs [79]. However, if specific microbiota abnormalities associated with adenomas and colon cancer progression—from healthy tissue to adenoma and adenoma to colon cancer—could be identified, it could enable early intervention and potentially improve the treatment, prognosis, and management of colon cancer [80,81,82].

#### 3.1.1. Butyric Acid and Short-Chain Fatty Acids (SCFAs)

Short-chain fatty acids (SCFAs), mainly acetic acid, propionic acid, and butyric acid, are products of bacterial metabolism in the colon. These compounds have multiple health benefits, including anti-inflammatory, anticancer, and intestinal barrier support properties. Butyric acid is produced primarily by anaerobic bacteria in the large intestine through the fermentation of non-digestible carbohydrates such as dietary fiber. The most important bacterial phyla responsible for butyric acid production include those listed below:

**Firmicutes**
  Firmicutes is one of the most abundant phyla in the human gut and comprises several families of butyric acid-producing bacteria, such as the following:
  - Ruminococcoccaceae: This family includes genera such as *Ruminococcus* and *Faecalibacterium*. *Faecalibacterium prausnitzii* is particularly notable for its ability to produce butyric acid and its anti-inflammatory properties.
  - Lachnospiracea: This includes genera such as *Butyrivibrio*, *Roseburia*, and *Anaerostipes*. *Anaerostipes* are known for their ability to ferment dietary fiber and produce butyric acid as the main end product.

**Bacteroidetes**

Although Bacteroidetes are not predominantly producers of butyric acid, their role in the degradation of complex polysaccharides provides the necessary substrates for butyric acid-producing bacteria in the phylum Firmicutes.

The symbiotic interaction between Bacteroidetes and Firmicutes is crucial for the efficient production of butyric acid in the colon.

#### 3.1.2. Protective Role of Short-Chain Fatty Acids (SCFAs)

**Anti-inflammatory Properties**

SCFAs, particularly butyric acid, have potent anti-inflammatory properties. Butyric acid can inhibit or activate the production of pro-inflammatory cytokines through the inhibition of the nuclear factor kappa-light-chain-enhancer of activated B cells (NF-κB) pathway. In addition, butyric acid promotes the differentiation of regulatory T cells (Treg), which play a crucial role in suppressing excessive immune responses.

**The Maintenance of the Intestinal Barrier**

Butyric acid is an important energy source for colonocytes (colon cells) and promotes intestinal barrier maintenance (colon cells) and the integrity of the intestinal barrier by increasing mucin production and strengthening the tight junctions between epithelial cells. This helps prevent the translocation of bacteria and toxins from the intestine into the bloodstream, reducing the risk of systemic inflammation and sepsis.

**Anticancer properties**

Butyric acid has significant anticarcinogenic effects. It induces apoptosis (programmed cell death) in colon cancer cells, arrests the cell cycle, and promotes cell differentiation. These effects are mediated through the inhibition of histone deacetylases (HDACs), leading to increased histone acetylation and the upregulation of gene expression, which promotes expression that promotes malignant cell death and normal cell differentiation.

#### 3.1.3. Microbiota and Chemotherapy

The intestinal microbiota plays a crucial role in modulating the pharmacokinetics and pharmacodynamics of various drugs used in cancer treatment. Intestinal microorganisms can influence the efficacy and toxicity of drugs through direct mechanisms, such as the biotransformation of compounds, and indirect mechanisms, by modulating the host’s immune system. For example, it has been shown that certain intestinal bacteria can metabolize chemotherapeutic drugs like irinotecan, affecting their bioavailability and efficacy. The microbiota converts irinotecan into a toxic metabolite, SN-38, which is glucuronidated in the liver and excreted in the bile. Intestinal bacteria deglucuronidate SN-38, increasing its reabsorption and intestinal toxicity [83].

The administration of antibiotics can significantly alter the composition of the intestinal microbiota, which, in turn, can modify the host’s response to chemotherapy. For example, the use of antibiotics has been shown to reduce the efficacy of 5-fluorouracil (5-FU) in the treatment of colorectal cancer by eliminating beneficial bacteria that metabolize this drug into active compounds or immune system modulators. A study revealed that patients treated with antibiotics had a lower response to 5-FU, suggesting that the microbiota plays a role in mediating the antitumor effects of this drug [84].

Additionally, chemotherapy-induced dysbiosis can lead to significant adverse effects, such as intestinal mucositis. Chemotherapy can cause an imbalance in the microbiota, decreasing commensal bacteria and increasing the proliferation of pathogens, which contributes to inflammation and damage to the intestinal epithelium. This effect not only affects the patient’s quality of life but also can influence the dosing and continuity of oncological treatment.

The use of probiotics and prebiotics is being investigated as a strategy to mitigate the adverse effects of chemotherapy through the modulation of the intestinal microbiota. Probiotics can help restore the microbial balance, improve intestinal barrier function, and reduce inflammation. A study demonstrated that the administration of probiotics in patients undergoing chemotherapy with irinotecan resulted in a significant reduction in treatment-induced diarrhea [85].

In summary, the interaction between the intestinal microbiota and drugs used in cancer treatment is complex and bidirectional. The microbiota can influence the efficacy and toxicity of chemotherapeutic drugs, while these drugs, in turn, can alter the microbial composition, leading to adverse effects. The use of antibiotics can further complicate this interaction, highlighting the need for personalized therapeutic approaches that consider the composition and function of the intestinal microbiota in cancer patients.

#### 3.1.4. Influence of Diet on Gut Microbiota and Its Impact on Carcinogenesis

Increasing evidence suggests that specific dietary components, such as fiber, fats, and proteins, significantly impact the composition and function of the gut microbiota, potentially having a substantial influence on and thereby relating to carcinogenesis. Recent studies have demonstrated that the gut microbiome responds rapidly and dynamically to dietary changes, in addition to cumulative long-term effects. This indicates that diet not only plays a critical role in shaping the microbial profile throughout life but also represents a modifiable factor with potential for intervention in cancer management strategies [86].

Studies comparing high-fiber, low-fat diets between African Americans and rural South Africans and Western low-fiber diets with high-fiber African diets have shown that high-fiber diets, when fermented by the gut microbiota, are associated with an increase in the production of short-chain fatty acids (SCFAs) such as butyrate. Butyrate has anti-inflammatory and anticancer properties, which can promote the apoptosis of cancer cells and reduce inflammation. The fermentation of fiber also lowers intestinal pH, creating a hostile environment for pathogenic and carcinogenic bacteria. Similarly, high-fiber diets were associated with greater microbiota diversity and reduced production of toxic metabolites.

High-fat diets, especially those rich in saturated fats, have been associated in various studies with a decrease in microbiota diversity, an increase in inflammation-associated bacteria, and a rise in the production of bile salts. These bile salts can be transformed by the microbiota into carcinogenic compounds, such as secondary bile acids, which damage DNA and contribute to the risk of colorectal cancer. They may also promote the growth of pro-inflammatory bacterial species, such as *Bilophila wadsworthia*, which is linked to inflammation and carcinogenesis [87,88].

Other studies indicate that a high-protein, low-carbohydrate diet results in the microbiota’s increased production, through protein fermentation, of harmful compounds such as heterocyclic amines, ammonia, sulfides, and phenols, which can be toxic and promote intestinal inflammation. Additionally, high protein intake has been observed to reduce microbial diversity and increase bacteria such as *Bacteroides* spp., which are associated with inflammatory diseases, and foster the growth of bacteria that metabolize these proteins into potentially carcinogenic compounds.

The ability of the intestinal microbiome to adapt and respond rapidly to dietary changes underscores its potential as a target in cancer therapeutic intervention. Modifying the diet to promote a beneficial microbiota can offer a complementary and effective approach to cancer prevention and treatment, integrating with other therapeutic modalities and becoming a valuable complement to conventional treatments. Therefore, the diet represents a crucial modifiable factor that can directly influence the intestinal microbiota and, consequently, cancer susceptibility and management.

#### 3.1.5. Impact of Environment on Gut Microbiota and Its Relation to Cancer

The environment, including exposure to environmental pollutants and lifestyles, plays a crucial role in shaping and modulating the gut microbiota, potentially significantly influencing the development and progression of cancer. Heavy metals such as lead, cadmium, and mercury, along with other industrial pollutants, can alter the composition and function of the gut microbiota and are associated with dysbiosis characterized by reduced bacterial diversity and an increase in potentially pathogenic bacteria. Dysbiosis may contribute to chronic inflammation, a risk factor for cancer development [89].

Chronic exposure to contaminants such as polycyclic aromatic hydrocarbons (PAHs) can induce alterations in the intestinal microbiota, increasing the prevalence of pro-inflammatory bacteria and reducing beneficial bacteria. This imbalance may contribute to the development of colorectal cancer through inflammatory and genotoxic mechanisms.

Pesticides and other agricultural chemicals, for example, glyphosate, a common herbicide, can also negatively impact the microbiota, including a decrease in the abundance of beneficial bacteria such as *Lactobacillus*. These changes can compromise the integrity of the intestinal mucosa, increase susceptibility to inflammatory diseases, and promote long-term carcinogenesis [90,91].

Similarly, lifestyles such as stress, sedentary living, and a lack of physical activity can negatively influence the intestinal microbiota, associated with changes in microbiota composition, including an increase in pathogenic bacteria such as Enterobacteriaceae. This can lead to increased intestinal permeability, allowing bacterial products to enter the systemic circulation and thereby promoting inflammation and potentially tumor development.

A lack of physical activity can also alter the intestinal microbiota, reducing microbial diversity and increasing the prevalence of pro-inflammatory bacteria, whereas regular physical activity is associated with a greater diversity of intestinal microbiota and an increased abundance of short-chain fatty acid-producing bacteria [92,93,94].

Other environmental factors, such as the excessive use of antibiotics, can cause severe dysbiosis by eliminating both pathogenic and beneficial bacteria, altering the composition and functionality of the microbiota, reducing microbial diversity, and increasing colonization by resistant bacteria. This imbalance can favor the development of inflammation and cancer by altering the production of protective bacterial metabolites and increasing susceptibility to colorectal carcinogenesis [94].

Understanding how these factors affect the microbiota can provide new opportunities to intervene in the management and prevention of cancer, emphasizing the importance of healthy environmental and lifestyle strategies.

### 3.2. Immunologically Mediated Relationship

#### 3.2.1. Positive Interactions between the Microbiome and the Immune System

The microbiome is instrumental in the development and maturation of the immune system. Shortly after birth, the gut microbiota begins to colonize the gastrointestinal tract, initiating critical immune responses. Commensal bacteria, such as *Bifidobacteria* and *Lactobacilli*, stimulate the production of regulatory T cells (Tregs), which are essential for maintaining immune tolerance and preventing excessive inflammatory responses [95].

The microbiota contributes to immune homeostasis by modulating the balance between pro-inflammatory and anti-inflammatory responses. Short-chain fatty acids (SCFAs), such as butyrate, produced by the microbial fermentation of dietary fiber, have anti-inflammatory properties. SCFAs bind to G-protein-coupled receptors (GPCRs) on immune cells, promoting the differentiation of Tregs and inhibiting the production of pro-inflammatory cytokines [95].

Tregs are fundamental in maintaining immune tolerance and preventing autoimmune responses, suggesting that butyrate may have a protective role in inflammatory and autoimmune diseases by increasing Treg populations in the intestine [85]. Additionally, SCFAs can influence the proliferation and activation of other lymphocyte cells, such as effector T cells and B cells. Butyric acid can inhibit the activation and proliferation of T cells by inhibiting histone deacetylases (HDACs), as mentioned in previous paragraphs, which modifies gene expression and reduces the production of pro-inflammatory cytokines. This epigenetic modulation also affects B cells, limiting their ability to proliferate and produce antibodies under inflammatory conditions.

The gut microbiota acts as a barrier against pathogenic microorganisms by competitive exclusion. Commensal bacteria outcompete pathogens for nutrients and attachment sites on the intestinal epithelium. Additionally, they produce antimicrobial peptides and stimulate the host’s production of mucins and defensins, which enhance the integrity of the gut barrier [96]. Nutrient competition involves commensal bacteria efficiently utilizing available nutrients, thereby depriving pathogens of essential resources necessary for their growth and colonization. For instance, certain commensal bacteria metabolize simple sugars and amino acids more efficiently than pathogens, limiting their proliferation. Metabolite competition involves the production of inhibitory substances, such as bacteriocins and short-chain fatty acids, which can directly inhibit pathogen growth. Additionally, the microbiota competes for adhesion sites on the intestinal epithelium, preventing pathogens from establishing a foothold. For example, *Lactobacillus* species can outcompete *Escherichia coli* for binding sites, thereby reducing the risk of infections

#### 3.2.2. Negative Interactions between the Microbiome and the Immune System

Dysbiosis, an imbalance in the microbial community, can lead to immune dysregulation and contribute to various diseases. For example, as stated above, a reduction in microbial diversity and an increase in pathogenic bacteria have been associated with inflammatory bowel diseases (IBDs), such as Crohn’s disease and ulcerative colitis. In these conditions, dysbiosis triggers excessive immune responses, leading to chronic inflammation and tissue damage [14].

Alterations in the gut microbiota composition can influence the development of autoimmune diseases. Certain bacterial species can mimic host antigens, leading to the activation of autoreactive T cells and the production of autoantibodies. For instance, specific strains of *Bacteroides* have been implicated in the pathogenesis of type 1 diabetes mellitus (T1DM), where molecular mimicry between bacterial antigens and pancreatic β-cell antigens triggers an autoimmune response [14].

The microbiome’s influence extends to allergic diseases such as asthma and atopic dermatitis. Early-life dysbiosis, often resulting from factors such as cesarean delivery, antibiotic use, and formula feeding, can impair the development of immune tolerance. This impairment leads to a higher risk of developing allergic diseases, characterized by T helper (Th) 2-skewed immune responses and elevated IgE levels [97].

## 4. Immune Mechanisms of Microbiome–Immune Interactions

The interactions between the microbiota and the host organism are, as we have seen so far, mediated by very diverse mechanisms. But, the fundamental interaction is with the immune system, since it is the immune system that must modulate the “size” and composition of this “organ” at any given moment. We are going to describe the aspects of the immune system that are relevant to this relationship and, above all, are linked to cancer, both its etiological and therapeutic aspects. We will focus on the mechanisms of immune activation blockade induced by the microbiota and on the so-called PD-1/PD-L1 axis, the basis of current immunotherapy, and the role of the microbiota in this aspect.

### 4.1. Pattern Recognition Receptors (PRRs)

The most immediate relationship of the microbiota with the host immune system is through the so-called innate (non-adaptive) immunity. Host immune cells express pattern recognition receptors (PRRs), such as Toll-like receptors (TLRs) and NOD-like receptors (NLRs), which recognize microbial-associated molecular patterns (MAMPs). The interaction between PRRs and MAMPs triggers signaling pathways that lead to the production of cytokines and chemokines, shaping the immune response. Commensal bacteria can modulate PRR signaling to promote tolerance and prevent excessive inflammation [98].

### 4.2. Immune Cell Modulation

A second, evolutionarily more modern branch of the immune system is the adaptive system. This not only has its own cells, such as B or T lymphocytes, but also incorporates the innate branch, providing it with more specificity and modulation points. We will review the essential ones for the modulation of the microbiota in relation to the antitumor response.

The microbiome influences the differentiation and function of various immune cells, including T cells, B cells, macrophages, and dendritic cells. For example, certain gut bacteria promote the differentiation of Th17 cells, which are important for mucosal immunity against bacterial and fungal infections. However, an overactive Th17 response can contribute to autoimmune and inflammatory diseases [99]. In the following section, we will go into this aspect in more detail.

### 4.3. Therapeutic Approaches against Cancer by Modulating the Microbiota and Immune System

Recent advancements in cancer research have identified the gut microbiota as a critical factor influencing cancer development and treatment outcomes. The microbiota, comprising trillions of microorganisms residing in the human gut, plays a pivotal role in modulating immune responses and maintaining metabolic homeostasis. Various strategies to manipulate the microbiota are being explored to enhance cancer therapies’ efficacy and mitigate adverse effects. Microbiota play essential roles in various physiological processes, including the development and function of the immune system. This review provides an overview of the emerging treatments focused on modulating the microbiota to treat cancer based on the current scientific literature. The Th17 lymphocyte subpopulation, which expresses the molecule RAR-related orphan receptor gamma T (RORγt), plays an essential role in the homeostasis of the microbiota.

RAR-related orphan receptor gamma T (RORγt) is a transcription factor belonging to the nuclear receptor superfamily. It is predominantly expressed in specific subsets of immune cells, including Th17 cells and innate lymphoid cells (ILCs). The protein plays a crucial role in the development, function, and regulation of these cells, particularly in the context of gut homeostasis and immunity, which have significant implications for cancer development and therapy [100].

### 4.4. RORγt in Microbiota and Cancer

Th17 cells, a subset of CD4+ T cells, are characterized by their production of interleukin-17 (IL-17). RORγt is the master regulator of Th17 cell differentiation. The presence of IL-6 and transforming growth factor-beta (TGF-β) drives the expression of RORγt, which then induces the transcription of IL-17 and other effector cytokines. These cytokines play vital roles in defending against extracellular pathogens, maintaining mucosal barriers, and modulating inflammatory responses [101,102]. RORγt is critical for the maintenance of immune homeostasis in the gut. It regulates the balance between pro-inflammatory and regulatory pathways to ensure tolerance to commensal microbiota while providing immunity against pathogens. High frequencies of both innate and adaptive immune cells expressing RORγt characterize the intestinal immune system, suggesting its importance in mediating responses to the microbiota [102].

The gut microbiota influences the differentiation and function of RORγt+ cells through various microbial metabolites. Short-chain fatty acids (SCFAs), derived from the fermentation of dietary fiber by gut bacteria, enhance RORγt expression and promote the differentiation of Th17 cells. This interaction is reciprocal, as RORγt+ cells also shape the composition of the gut microbiota, promoting a symbiotic relationship that is essential for gut health [103].

The dual role of Th17 cells in cancer is complex and context-dependent. RORγt+ Th17 cells can enhance antitumor immunity by recruiting and activating cytotoxic T cells and other immune effector cells in the tumor microenvironment. However, Th17 cells can also promote tumor progression and metastasis through chronic inflammation and immunosuppression. The balance of these effects is influenced by the local cytokine milieu, the presence of specific microbial species, and the overall immune context of the host [103].

In summary, RORγt serves as a crucial link between the microbiota and the immune system, with significant implications for cancer biology. Its regulation of Th17 cells and interaction with the gut microbiota underscore its importance in maintaining immune homeostasis and highlight its potential as a therapeutic target in cancer immunotherapy.

## 5. Other Mechanisms of Microbiome-Mediated Immune Modulation in Cancer

A balanced microbiome contributes to the maintenance of immune surveillance, which is the immune system’s ability to detect and destroy malignant cells. Certain gut bacteria can enhance the efficacy of immune surveillance by promoting the activation and proliferation of cytotoxic T cells and natural killer (NK) cells. For instance, *Bifidobacterium* and *Lactobacillus* species have been shown to enhance the antitumor activity of CD8+ T cells [93]. A reduction in beneficial bacteria and an overgrowth of pathogenic microbes can lead to chronic inflammation, immune dysregulation, and a favorable environment for tumorigenesis. For example, increased levels of *Fusobacterium Nucleatum* have been associated with colorectal cancer [74], where it can inhibit the antitumor immune response and promote tumor growth [104].

## 6. The Role of the Microbiota in Modulating PD-1/PD-L1 Expression and Activation

The gut microbiota influences the immune system’s function, which is crucial for effective anticancer responses. Beneficial bacteria can enhance the activation and proliferation of cytotoxic T cells, which are essential for targeting and destroying cancer cells. By modulating the gut microbiota, it is possible to improve the efficacy of immune checkpoint inhibitors (ICIs) such as anti-PD-1 and anti-CTLA-4 antibodies. Studies have shown that a diverse and balanced microbiota is associated with better responses to ICIs, highlighting the potential of microbiota modulation in immunotherapy [105,106,107]. The gut microbiota interacts with the immune system by modulating the activity of various immune cells. For instance, certain gut bacteria can enhance the activation and proliferation of cytotoxic T cells, which are critical for antitumor immunity. The interaction between the microbiota and the immune system is mediated through pattern recognition receptors (PRRs), such as Toll-like receptors (TLRs), which recognize microbial-associated molecular patterns (MAMPs) and initiate immune responses [98].

**Figure 2:**
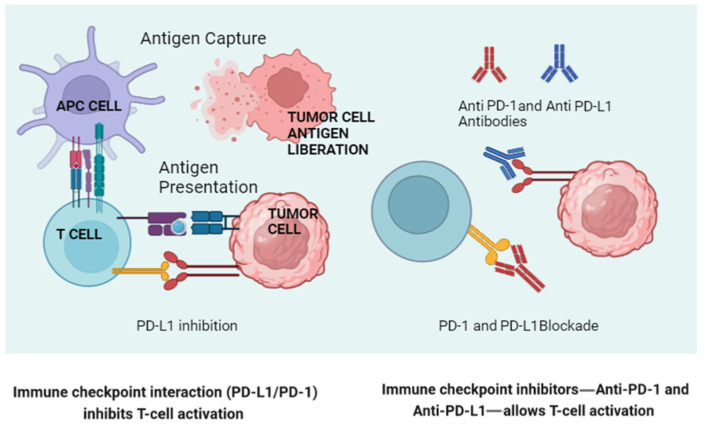
A schematic representation of normal lymphocyte activation via antigenic presentation (left), illustrating how a tumor cell blocks this activation through engagement of the PD-1/PD-L1 axis engagement. On the right, the way in which the new monoclonal antibodies block this “inactivation” is shown. (Created in BioRender.com.) APC: Antigen-Presenting Cell.

The gut microbiota can influence the expression of PD-1 and PD-L1 on immune cells and tumor cells (Figure 2). Studies have shown that certain gut bacteria can modulate the expression of these molecules, thereby affecting the efficacy of immune checkpoint blockade therapies. For example, *Bifidobacterium* species have been associated with enhanced antitumor responses and the increased efficacy of anti-PD-L1 therapy. These bacteria can modulate the tumor microenvironment, reducing the expression of immunosuppressive molecules and enhancing T-cell infiltration.

On the other hand, *Bifidobacterium* enhances dendritic cell maturation and subsequent T-cell activation, increasing the antitumor immune response. *Akkermansia muciniphila* has been shown to restore the efficacy of PD-1 blockade in antibiotic-treated or germ-free mice by inducing IL-12 production, which promotes T helper 1 (Th1) responses and improves cytotoxic T lymphocyte (CTL) activity.

Metabolite production by the gut microbiota, particularly short-chain fatty acids (SCFAs) like butyrate, acetate, and propionate, as mentioned in previous paragraphs, also plays a crucial role. SCFAs can modulate the immune response by promoting the differentiation and function of regulatory T cells (Tregs), reducing inflammation, and enhancing the gut barrier function. Furthermore, the production of microbial metabolites such as inosine and polyamines has been linked to enhanced T-cell responses and improved outcomes in cancer immunotherapy. The presence of specific bacterial species and their metabolites thus creates an immunomodulatory environment that can significantly influence the efficacy of PD-1/PD-L1 inhibitors, highlighting the importance of considering gut microbiota composition and function in optimizing immunotherapeutic strategies [108].

The gut microbiota can also affect the overall immune landscape, influencing the outcome of PD-1/PD-L1 blockade therapies. Microbial diversity and composition are crucial factors in determining the immune system’s response to these therapies. Patients with a higher diversity of beneficial gut bacteria tend to respond better to immune checkpoint inhibitors. This is because a diverse microbiota can enhance the activation and proliferation of antitumor T cells, leading to more robust immune responses [109].

## 7. Clinical Implications and Therapeutic Strategies

### 7.1. Enhancing Immunotherapy with Probiotics

Given the significant influence of the gut microbiota on the efficacy of anti-PD-1 and anti-PD-L1 therapies, there is growing interest in using probiotics to enhance treatment outcomes. Probiotics, which are live beneficial bacteria, can help restore a healthy microbial balance and improve the immune system’s ability to fight cancer. Clinical trials are underway to evaluate the potential of probiotic supplementation in combination with immune checkpoint inhibitors [110].

### 7.2. Fecal Microbiota Transplantation (FMT)

Fecal microbiota transplantation (FMT) involves transferring fecal matter from a healthy donor to a patient to restore a balanced gut microbiota. FMT has shown promise in enhancing the efficacy of cancer immunotherapy. Studies have demonstrated that FMT can improve the response to PD-1 blockade in patients with refractory melanoma. This suggests that manipulating the gut microbiota can be a powerful strategy to enhance the effectiveness of immune checkpoint inhibitors [111].

## 8. Discussion and Future Directions

The concept of precision microbiome modulation involves tailoring interventions based on an individual’s microbiome profile to optimize treatment outcomes. This approach requires a deep understanding of the specific microbial species and their functions that are associated with favorable responses to PD-1/PD-L1 blockade. Advanced sequencing technologies and bioinformatics tools are being used to identify microbial biomarkers that can predict treatment responses and guide personalized therapies. 

Due to its pivotal role in regulating gut immunity and its involvement in cancer, RORγt represents a potential therapeutic target. Modulating RORγt activity could potentially enhance antitumor responses and restore gut homeostasis. For instance, agonists or antagonists of RORγt could be developed to either promote its function in cases of immune deficiency or inhibit its activity to reduce pathological inflammation and cancer progression. However, therapeutic strategies need to be carefully designed to avoid disrupting the delicate balance of gut homeostasis and immune regulation.

Longitudinal studies are essential to understanding the dynamic interactions between the gut microbiota and the immune system over time. These studies can provide insights into how changes in the microbiota influence the expression and activation of PD-1 and PD-L1 during cancer progression and treatment. Such knowledge can inform the development of strategies to modulate the microbiota at different stages of cancer therapy to maximize treatment efficacy. Numerous clinical trials are investigating the role of microbiota manipulation in cancer therapy. These trials are exploring various interventions, including probiotic supplementation, prebiotic dietary interventions, FMT, and microbiota-targeted drugs. For instance, a clinical trial is evaluating the impact of FMT on the efficacy of anti-PD-1 therapy in metastatic melanoma patients. Another trial is assessing the effects of a probiotic cocktail on the immune response in patients undergoing chemotherapy. These studies aim to establish the efficacy and safety of microbiota modulation strategies in enhancing cancer treatment outcomes.

Some bacteria, such as *Fusobacterium*, appear to be implicated in tumor development, whereas others, like *Bifidobacterium*, exhibit a protective effect against cancer by stimulating the immune system, including T cells and macrophages. Moreover, they demonstrate the potential to reduce tumor growth and are linked to favorable responses to chemotherapy or immunotherapy. Conversely, certain types of intestinal flora are associated with poor treatment responses.

The identification of these associations paves the way for future research and therapeutic approaches, such as fecal transplantation or the utilization of the microbiota as a potential biomarker for treatment response.

## 9. Conclusions

There is growing evidence from not only preclinical but also clinical trials that the microbiota plays an essential role in modulating both innate and adaptive antitumor immune responses. With the new therapeutic strategies in solid tumors based on the PD1-PDL1 axis, it is essential to know the role of the microbiota in both the eventual potentiation and inhibition of the therapeutic effect. We know that in hematological tumors in preclinical studies with Chimeric Antigen Receptor T-cells (CART-T cells), it is possible to modulate the immune responses of these cells with antibiotics. More clinical trials are needed to establish the most appropriate methodology to obtain the maximum benefit from conventional treatments (chemotherapy) and, mainly, immunotherapy.

## 10. Search Strategy

A search in Google Scholar and PubMed was carried out for this review. The following search terms were used: microbiota and cancer, microbiota modulation, microbiota immune modulation, chemotherapy and microbiota, microbiota modulation strategies, PD-1/PDL-1 and microbiota, and prebiotics and probiotics. The most relevant ones were chosen in “the role of the microbiota in the modulation of the antitumor immune response”. The chosen period was between 2011 and 2023.

## Figures and Tables

**Table 1:** Intestinal microbiota composition: main phyla and genera.

Bacterial Population	Description	Highlighted Genera	Functions
**Firmicutes**	One of the most abundant populations in the intestine, representing around 60–70% of the total microbiota.	*Lactobacillus*, *Clostridium*, *Enterococcus*, *Ruminococcus*	Fermentation of indigestible carbohydrates, production of SCFA, modulation of the immune system.
**Bacteroidetes**	Constitutes approximately 20–30% of the intestinal microbiota.	*Bacteroides*, *Prevotella*, *Porphyromonas*	Degradation of complex polysaccharides and dietary fibers, production of SCFA, regulation of lipid metabolism and energy homeostasis.
**Actinobacteria**	Less abundant but important components of the intestinal microbiota.	*Bifidobacterium*, *Corynebacterium*, *Propionibacterium*	Fermentation of carbohydrates, production of lactic and acetic acid, inhibition of pathogen growth.
**Proteobacteria**	Constitutes a minority under healthy conditions but can significantly increase in dysbiosis.	*Escherichia*, *Salmonella*, *Helicobacter*, *Vibrio*	Some species are normal commensals, while others can be pathogenic and cause gastrointestinal diseases.
**Verrucomicrobia**	Represented mainly by a single genus in the human intestine.	*Akkermansia*	Degradation of mucin, beneficial effects on metabolic health and intestinal barrier integrity.
**Fusobacteria**	Less prevalent but present in the intestinal microbiota.	*Fusobacterium*	Biofilm formation, potential pathogenesis of gastrointestinal diseases.
**Cyanobacteria**	A group of bacteria that obtain their energy through photosynthesis and are found in various environments including the human intestine.	*Prochlorococcus*, *Synechococcus*	Photosynthesis, nitrogen fixation, potential interactions with gut flora.

**Table 2:** Bacteria and viruses involved in human cancer (modified from ref. [11] *Oncotarget* 2018 Apr 3; 9 (25), 17915–17927).

	Cancer
*Human papillomavirus*	Oro-pharyngeal carcinoma Anogenital carcinoma
*Epstein–Barr virus*	Naso-pharyngeal carcinoma Lymphoma
*Helicobacter pylori*	Esophageal adenocarcinoma Gastric adenocarcinoma Gastric lymphoma
*Hepatitis B virus*	Hepatocellular carcinoma
*Hepatitis C virus*	Hepatocellular carcinoma
*Human immunodeficiency virus*	Lymphoma
*Human herpes virus 8*	Karposi sarcoma
*Human T-cell lymphotropic virus type I*	Adult T-cell leukemia
